# Large-Scale Monitoring and Risk Assessment of Metal Contamination from Urban Areas in the Amazon River Basin

**DOI:** 10.1007/s00244-026-01206-4

**Published:** 2026-07-02

**Authors:** Alexeia Barufatti, Andrea V. Waichman, Rhaul de Oliveira, Isabel López-Heras, Marco Vighi, Andreu Rico

**Affiliations:** 1 Faculdade de Ciencias Biologicas e Ambientais (FCBA), Universidade Federal da Grande Dourados (UFGD), Rod. Dourados Itahum km 12., Dourados, MS 79804‑970 Brazil; 2 Institute of Biological Sciences, Federal University of the Amazon, Av. Rodrigo Ramos 3000, Manaus, 69077-000 Brazil; 3 School of Technology Rua Paschoal Marmo, University of Campinas (Unicamp), 1888 – Jd. Nova Itália, Limeira, SP 13484-332 Brazil; 4 IMDEA Water Institute, Science and Technology Campus of the University of Alcalá, Av. Punto Com 2, Alcalá de Henares, 28805 Madrid, Spain; 5 Cavanilles Institute of Biodiversity and Evolutionary Biology, University of Valencia, c/ Catedrático José Beltrán 2, 46980 Paterna, Valencia, Spain

## Abstract

In this study we provide a comprehensive assessment of metal contamination in the Amazon River and its main tributaries, with a focus on urban contamination hotspots. A monitoring campaign was conducted across 40 sampling sites, analysing water concentrations of As, Cd, Cu, Cr, Fe, Mn, Pb, Ni, and Zn. Metal concentrations were evaluated against national and international water quality standards and compared between urbanized and less impacted regions to delineate pollution patterns. Additionally, an ecological risk assessment was conducted for individual metals and for metal mixtures using Species Sensitivity Distributions (SSDs). The results of this study show that Fe levels in the Amazon River were exceptionally high, with potential implications for aquatic species distribution across the basin. Exceedances of water quality standards were identified for four metals (Fe, Pb, Mn, Cu), with metal enrichment observed particularly in Manaus, Macapá, and Belém. Acute multi-substance potentially affected fraction (msPAF) of species in the Amazon River and its tributaries indicated insignificant risks, while chronic values ranged from 3% to 33%. In urban areas, acute msPAF values were generally low, with the exception of two samples taken in Manaus that exhibited values of 11% and 16%. Chronic riskvalues, however, were consistently high in these urban locations, reaching up to 72% of species potentially affected and reflecting sustained ecotoxicological stress. Cu, Zn and Mn were identified as the primary contributors to chronic toxicity, with Cu and Zn dominating in Manaus, and Mn in Macapá. These findings underscore the need for stricter industrial discharge regulations and improved wastewater treatment systems to preserve Amazonian freshwater ecosystems.

**Supplementary Information:**

The online version contains supplementary material available at https://doi.org/10.1007/s00244-026-01206-4.

## Introduction

Metal contamination is increasingly recognized as a significant threat to aquatic ecosystems worldwide (Niu et al. 2021). Anthropogenic activities, including industrial runoff, untreated urban wastewater, and mining activities, are among the primary sources of metal contamination in surface waters (Azimi et al. 2017; Macklin et al. 2023). Additionally, agricultural runoff and intensive livestock farming significantly contribute to metal loading through the application of fertilizers and pesticides containing trace metals, as well as the accumulation and subsequent mobilization of livestock waste into adjacent water bodies (Rashid et al. 2023).

Metals can exert acute and chronic toxicity to aquatic organisms and can bioaccumulate in trophic food webs, posing substantial risks to both ecosystem integrity and human health (de Paiva Magalhães et al. 2015; Tchounwou et al. 2012). While there is a wealth of metal contamination data and risk assessment studies for major rivers in developed regions such as Europe and North America (Niu et al. 2021, Topa et al. 2024), comprehensive data for large rivers in tropical regions remain scarce.

The Amazon River, the largest river in the world, contributes approximately one-third of the total river water discharge into the oceans. Despite its significance, there is limited information about its overall chemical status, particularly concerning metal contamination. Only a limited number of studies provide monitoring data for certain tributaries of the Amazon River, with most focusing on mining activities as the main source of metal contamination (Moulatlet et al. 2023; Santana et al. 2007; Seyler and Boaventura 2001, 2003).

The emission of untreated wastewater discharges from urban areas has been increasingly acknowledged as another important source of metal contamination into the Amazon River (Ferreira et al. 2020). This threat is critical due to the increasing urban population and the lack of adequate sanitation and wastewater treatment facilities (Gomes et al. 2023). According to a survey carried out by Instituto Trata Brasil (2022), approximately 90% of the population in northern Brazil lacks access to sewage collection services. In line with this, previous studies have revealed that aquatic ecosystems impacted by wastewater from urban areas of the Amazon region contain high levels of potentially toxic compounds such as pharmaceuticals, pesticides, polycyclic aromatic hydrocarbons or microplastics, which contribute to ecosystem deterioration and the loss of regional biodiversity (Rico et al. 2021, 2022, 2023; Rizzi et al. 2023). Further, a meta-analysis of water and sediment samples collected between 1990 and 2023 found that 56% of the water samples contained metal concentrations exceeding ecotoxicological thresholds, with the most significant exceedances being found near large cities characterized by intense industrial activity (Ferreira et al. 2020). Therefore, previous investigations highlight a need for careful monitoring of metal contamination in urban areas of the Amazon region and emphasize the importance of further research to better understand the ecological impacts of untreated urban wastewater discharges across the basin.

This study provides a comprehensive assessment of metal contamination in the Amazon River and its primary tributaries, with focus on major urban areas as potential contamination hotspots. We analysed metal concentrations across 40 sites, focusing on arsenic (As) (metalloid), cadmium (Cd), chromium (Cr), copper (Cu), iron (Fe), manganese (Mn), nickel (Ni), lead (Pb), and zinc (Zn). The specific objectives of this study were (1) to characterize metal contamination in the Amazon River and its main tributaries, including streams across the major urban areas of the Brazilian Amazon, (2) to compare the exposure concentrations with the existing national and international water quality standards, and (3) to assess the ecological risks posed by metal mixtures using a probabilistic risk assessment approach. The findings of this study contribute to a better understanding of the spatial distribution of metal contamination across the Amazon River basin and its potential ecological implications, providing critical insights for water quality monitoring and conservation.

## Materials and Methods

### Field Sampling

**Fig. 1:**
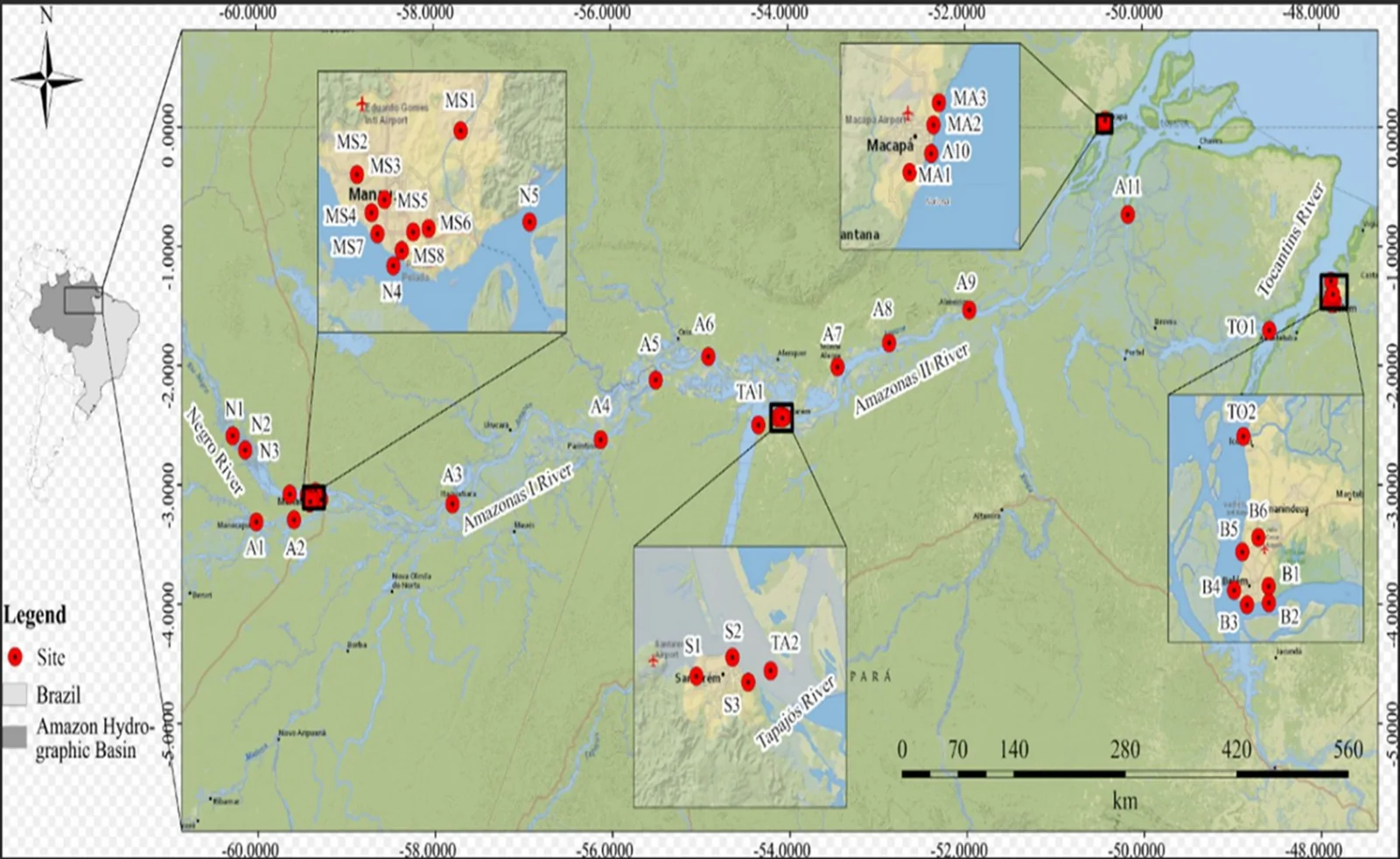
Location of sampling sites in the Amazon River I (A1–A6) and II (A7–A11); in its main tributaries: Negro River (N1–N5), Tapajós River (TA1–TA2), Tocantins River (TO1–TO2); and in the urban areas of Manaus (MS1–MS8), Santarém (S1–S3), Macapá (M1–M3) and Belém (B1–B6). For further details regarding the sampling locations are provided in the Supplementary Material (Table S1)

A field sampling campaign was conducted between November and December 2019, during the dry season, covering a distance over 1,500 km. Water samples were collected from 40 sites, strategically selected to encompass both major urban areas and less impacted regions of the Brazilian Amazon. Sampling sites included 11 locations along the Amazon River, categorized into Amazon I (A1–A6) and Amazon II (A7–A11), based on their geographical distribution (Fig. 1). Additional samples were taken from three major tributaries: Negro River (N1–N5), Tapajós River (TA1, TA2), and Tocantins River (TO1, TO2). Furthermore, smaller tributaries and streams crossing the urban areas of Manaus (MS1–MS8), Santarém (S1–S3), Macapá (M1–M3), and Belém (B1–B6) were sampled (Fig. 1).

To capture the spatial heterogeneity of metal contamination, specific sites were selected based on proximity to urban discharges, industrial activities, and taking into account hydrodynamic characteristics. In the Negro River, the sampling sites N1–N2 were located within the Anavilhanas National Park, serving as reference sites with minimal anthropogenic influence, while N4 and N5 were positioned at the urban discharge plume of Manaus. In the Amazon River, sampling sites were distributed near smaller urban settlements and harbour zones to assess potential pollution sources. In Belém, B1–B4 were strategically located at the discharge points of minor tributaries that flow into the Tocantins River, aiming at assessing worst-case urban effluent impacts. Sampling sites in Macapá, Tocantins River, and Belém were subjected to tidal effects, which can impact metal dispersion and dilution. To minimize variability associated with tidal mixing, all samples in these regions were collected during low tide, ensuring a more consistent assessment of metal concentrations across sites.

Water samples were collected using a stainless-steel bucket that was pre-rinsed with river water several times before the sample was collected. Sampling was conducted either from boats or urban bridges, ensuring collection at a standardized depth of 20–30 cm below the water surface to minimize surface water interference. Immediately after collection, 250 mL water aliquots were transferred into high-density polyethylene (HDPE) bottles, previously rinsed two times with river water. To ensure sample stability and prevent metal precipitation, 69% (v/v, trace analysis grade) nitric acid (HNO_3_) purchased from Scharlau (Barcelona, Spain) was added to adjust the pH to 2. Acidification was done in situ because it was not possible to access a suitable water chemistry laboratory for filtration and processing until several days after sampling. Therefore, this approach was considered suitable to ensure sample stability and to prevent metal precipitation, microbial activity, and redox changes. All samples were sealed and stored at room temperature (~ 25 °C) until further processing and analysis.

### Metal Analysis

#### Chemicals and Standards

All reagents used in the analyses were certified analytical grade. Nitric acid (65% v/v) and individual ICP-MS (Inductively Coupled Plasma Mass Spectrometry) standard solutions, each containing 1000 mg L^− 1^ of As, Cd, Cr, Cu, Fe, Mn, Ni, Pb and Zn, were purchased from Fluka (St. Louis, USA). A multi-element standard stock solution (10 mg L^− 1^) was prepared using ultrapure water (18.2 MΩ·cm) obtained from a Millipore Milli-Q water purification system (Darmstadt, Germany) and 1% (v/v) nitric acid by diluting individual ICP-MS standard solutions. Working standards for the external calibration method were prepared daily by further dilution. In addition, an internal standard solution (10 mg L^− 1^) containing Li, Sc, Ge, Y, In, Tb, and Bi, and a tuning solution (10 mg L^− 1^) containing Ce, Co, Li, Mg, Tl, and Y, were purchased from Agilent Technologies (Santa Clara, USA).

#### Analysis of Metals by ICP-MS

Two aliquots of 80 mL were separated for the analysis. The first one was filtered through a 0.45 μm syringe filters (Vidrafoc, Barcelona, Spain) for the analysis of the recoverable fraction (or acid-extractable fraction), which consists of dissolved metals plus metals weakly bound to suspended particles and released after the field acidification. The second was subjected to microwave digestion at 190 °C for 30 min using an Ethos One Microwave Digestion System (Milestone, Bergamo, Italy) with a mixture of HNO_3_:H_2_O_2_ (4:1), and then filtered through the same syringe filters, for the analysis of the total metal concentration in the sample.

The analysis of metals was performed using a 7700 Inductively Coupled Plasma Mass Spectrometry (ICP-MS) system equipped with a MicroMist concentric nebulizer from Agilent Technologies (Palo Alto, CA, USA). The plasma conditions were as follows: forward power of 1550 W, plasma gas flow rate of 15 L/min, auxiliary gas flow rate of 0.9 L/min, collision gas (He) flow rate of 4.3 L/min, and nebulizer gas flow rate of 1.1 L/min. Most of elements were quantified and confirmed with the recording of the 2 isotopes, except for As and Mn (see Table S2 for further details).

Calibration curves were constructed using the mean signal obtained from three replicate injections for each calibration level. Linear regressions showed coefficients of determination (R^2^) higher than 0.99. Calibration was performed in 1% v/v HNO_3_ over a concentration range of 0.005–2000 µg L^−1^ for iron (Fe) and 0.005–300 µg L^−1^ for the remaining elements (As, Cd, Cr, Cu, Mn, Ni, Pb, and Zn).

Limits of detection (LODs) and limits of quantification (LOQs) were calculated from the ICP-MS signal of analytical blanks, expressed as counts per second (cps), measured throughout the analytical sequence. LODs and LOQs were calculated as the mean blank signal plus three and ten times its standard deviation, respectively. The resulting signal-based thresholds were converted into concentration units using the corresponding calibration curves. The LOD and LOQ values obtained for each element are provided in Table S3.

Instrumental precision was assessed as the relative standard deviation of three consecutive injections of each sample, with RSD values ≤ 15%, and possible variations on instrumental response were controlled by the on-line addition of an internal standard solution at 400 µg L^−1^. To ensure analytical reliability, samples were analyzed at different dilution factors (1:5 and 1:50). Analytical blanks and procedural blanks, the latter for digested samples, were injected every three samples. In addition, a quality control solution containing the target elements at 50 µg L^−1^ was analyzed throughout the analytical sequences. Accuracy was evaluated through recovery experiments performed in water samples (*n* = 6) spiked with a mixed standard solution at different concentration levels selected according to the basal concentrations of the target elements (1, 10, 50, 100, and 1000 µg L^−1^). Recovery values ranged from 89 to 104% for samples taken in the Amazon river and tributaries, and from 90 to 101% for samples taken in areas with urban impact, with relative standard deviations below 10% (Table S4).

### Metal Exposure Assessment

The metal exposure levels of the different samples was evaluated in relation to the sample groups, which account for different metal contamination sources. A Redundancy Analysis (RDA) with 999 Monte Carlo permutations was performed using Canoco 5.0 software (ter Braak and Šmilauer, 2012) to visualize variations in the metal fingerprint across sample groups. Prior to the analysis, data were log-transformed using the log (x + 1) transformation to reduce the influence of extreme values and improve normality. Metal concentrations below the limit of detection (LOD) were transformed to LOD/2, while metals detected below the limit of quantification (LOQ) were assigned to the midpoint between the LOD and LOQ.

Additionally, we calculated metal Enrichment Factors (EFs), to determine whether metal concentrations are within natural levels or come from antrophogenic activities:

1 $$\:EF=\:\frac{{\left({C}_{metal}/{C}_{reference}\right)}_{sample}}{{\left({C}_{metal}/{C}_{reference}\right)}_{background}}$$

where, C_metal_ is the total concentration of the target metal, C_reference_ is the total concentration of a reference metal that is naturally abundant and is not expected to be significantly influenced by human activities. In this case Fe was selected as reference. The enumerator correponds to the ratio betweeen the C_metal_ and C_reference_ in the evaluated sample, while the denominator correponds to the ratio between the C_metal_ and C_reference_ in a natural, unpolluted location. In this study, the mean of the total concentrations measured in the Anavilhanas National Park (N1 and N2) were used as background. Samples with metals having EFs below 1.5 were considered to have minimal enrichment; samples with EFs between 2 and 5 were considered to have moderate enrichment (i.e., characteristic of industrial emissions and/or urban runoff); and samples with EFs above 5 as having very high enrichment (i.e., strong pollution signal).

### Comparison with Regulatory Standards

The measured exposure concentrations were compared with the water quality standards set by CONAMA 357/2005 (Brasil, 2005) and the EU Directive 2013/39/EU (European Comission 2013). The CONAMA 357/2005 regulation provides standards for all the metals included in this study for different water uses. The standards selected for this study were the ones corresponding to Class 1 freshwater bodies, which are designated for multiple uses, including human consumption, the protection of aquatic communities, recreational activities, agricultural irrigation, and the protection of aquatic environments in Indigenous Territories. The EU Directive 2013/39/EU sets maximum allowable concentrations and annual average water quality standards for three metals: Cd, Ni and Pb.

### Ecological Risk Assessment

Acute and chronic toxicity data for primary producers, invertebrates, and fish were collected from the US Environmental Protection Agency (2024) ECOTOX database for the nine measured metals using the CAS numbers listed in Table S5. Freshwater toxicity data for different taxonomic groups included laboratory studies published between 1998 and 2023, except for Fe and Mn, whose publication periods were expanded to 1985–1986 due to the paucity of studies. The collected toxicity data were restricted to the endpoints, measured effects, and test exposure durations listed in Table 1. When multiple toxicity values were found for a given measured effect or exposure duration, the geometric mean was calculated. For the same taxon, when multiple exposure durations or measured effects were available, the lowest toxicity value was selected. The acute and chronic toxicity data used in this study are shown in Tables S6 and S7, respectively.

**Table 1:** Criteria for the selection of the toxicity data included in this study.

	Fish		Invertebrates		Primary producers
	Acute	Chronic	Acute	Chronic	Chronic
End point	LC_50_	NOEC or EC_10_	EC_50_	NOEC or EC_10_	EC_50_/EC_10_ or NOEC
Measured effect	Mortality	Growth, development, behavior, mortality	Mortality, behavior	Growth, behavior, mortality, reproduction	Growth
Test duration	2 to 4 d	> 21–100 d	2 to 4 d	> 7 to 100 d	3 to 15 d

LC_50_: median lethal concentration; NOEC = no-observed effect concentration. EC_10_: concentration that affects to 10% of organisms

Ecological risks were calculated based on the Potentially Affected Fraction (PAF) of species using Species Sensitivity Distributions (SSDs; Posthuma et al. 2002) built with acute and chronic toxicity data (Table S6 and S7). SSDs were built using a log-normal distribution and the ETX 2.3 software (Van Vlaardingen et al. 2005) considering at least 8 data points. The calculated SSD parameters for each metal, including the HC5 and the HC50 (i.e., Hazardous Concentration for the 5% and 50% of species, respectively) and the standard deviation (σ), are shown in Table 2.

The PAF of species by a single metal and the multi-substance Potentially Affected Fraction (msPAF) were calculated to assess the risks posed by single metals and for metal mixtures contained in the different samples, respectively (de Zwart and Posthuma 2005). The PAF was calculated based on the SSD parameters shown in Table 2 and using recoverable metal concentrations. The msPAF was calculated following the methods described in Rico et al. (2021) assuming independent action.

The hazardous concentration for 5% of species (i.e., resulting in a protection level of 95% of species) is commonly used as a concentration threshold to benchmark unacceptable ecological effects in chemical risk assessment (Posthuma et al. 2002). In our study, sampling sites with single compound PAF or msPAF values above this threshold were considered to be potentially impacted by the measured metal concentrations.

**Table 2:** Species Sensitivity Distribution (SSD) parameters for acute and chronic exposure.

	Acute SSD parameters				Chronic SSD parameters			
	*n*	HC5 (LL-UP)	HC50 (LL-UP)	σ	*n*	HC5 (LL-UP)	HC50 (LL-UP)	σ
As	8	90 (5.4–387)	2437 (673–8818)	0.8	9	2.3 (0.04–20)	383(60–2437)	1.3
Cd	32	7 (2–17)	342 (169–689)	1.2	17	0.03(0.002–0.1)	8 (2–31)	1.4
Cr	10	56 (2.9–292)	3485 (849–14310)	1.0	8	4 (0.09–28)	341(60–1925)	1
Cu	105	9.6 (0.5–15)	240 (175–330)	0.8	49	0.8 (0.3–1.6)	32 (19–56)	0.9
Fe	28	72 (14–238)	7479 (3052–18330)	1.2	8	85 (10–256)	1053 (389–2700)	0.6
Mn	8	2392 (383–6087)	19,740 (8672–44940)	0.5	8	71 (9–202)	749(300–1871)	0.6
Ni	9	202 (11–978)	8542 (2199–33180)	0.9	8	5(0.4–17)	84(28–249)	0.7
Pb	11	34(1.5–212)	3853 (841–17680)	1.2	14	1.8(0.17–8.2)	138 (40–465)	1
Zn	55	78 (38–139)	1652 (1090–2502)	0.7	17	9.7(1.8–29)	318 (131–767)	0.9

n: number of toxicity values used to construct the SSD; HC5 (LL-UL): Hazardous Concentration for 5% of species together with the lower and upper limit of the 90% confidence interval in µg/L; HC50: Hazardous Concentration for 50% of species together with the lower and upper limit of the 90% confidence interval in µg/L; σ: standard deviation

## Results and Discussion

### Metal Exposure Assessment

**Fig. 2:**
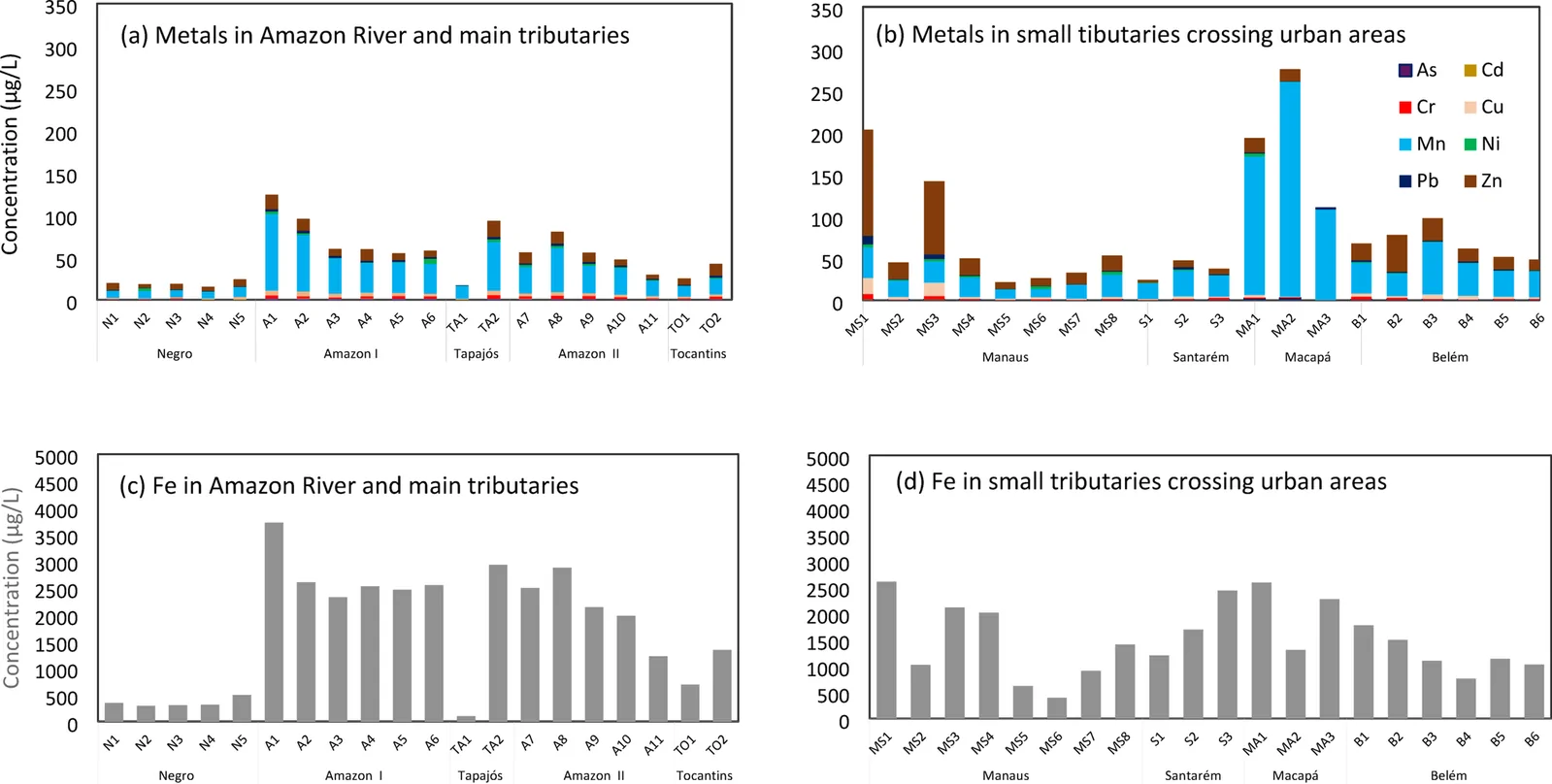
Total concentrations of metals (excluding Fe) in (**a**) the Amazon River and main tributaries, and (**b**) in small streams and tributaries crossing urban areas. Total Fe concentrations in (**c**) the Amazon River and main tributaries, and (**d**) in small streams and tributaries crossing urban areas

The results of the recoverable and total metal concentrations in the analyzed samples are summarized in Table 3. Regarding the Amazon River and its main tributaries, samples from the Negro River and upstream areas of the Tapajós River exhibited significantly lower total metal concentrations (Table 3; Fig. 2). In contrast, samples collected from the Amazon River (sites I and II) contained the highest concentrations of Fe and Mn compared to other tributaries. Total Fe concentrations in these samples ranged from 1,218 to 3,719 µg/L, while total Mn concentrations ranged between 18 and 90 µg/L. In the Amazon tributaries (excluding sample TA2, taken at the confluence of the Tapajós and Amazon rivers), total Fe concentrations ranged from 293 to 1,339 µg/L, and total Mn concentrations from 8 to 36 µg/L.

Our findings suggest that total metal exposure in the Amazon River and its tributaries varies based on the basin’s geochemistry, with lower metal concentrations in key tributaries (Negro River, Tapajós River, Tocantins River) and elevated Fe and Mn levels in the samples taken in the Amazon River (I and II). This variation likely results from geological and sedimentary processes influenced by the Andean uplift, which enriches sediments and waters flowing towards the main Amazon River with metals from the Andes region (Furch and Junk 1997; Hoorn et al. 2010). These metals are abundant in Amazon soils, primarily composed of red ferralitic soils (Quesada et al. 2010; Sombroek 2000). The mineralogy of these soils is dominated by quartz, Al and Fe oxides, and kaolinite (Seyler and Boaventura 2003). As noted by Souza et al. (2018), ferralitic soils retain the geochemical signatures of their parent materials, even under intense weathering, and are characterized by Fe and Al oxides, high clay content, and strong acidic weathering.

In urban areas, cumulative metal concentrations were generally highest in Macapá, followed by Manaus, Belém, and Santarém (Fig. 2). Macapá’s cumulative metal concentrations (excluding Fe) were dominated by Mn, ranging from 108 to 256 µg/L. In contrast, samples from Manaus revealed Zn as the dominant metal, followed by Mn and Cu. Maximum total Zn concentrations reached 128 µg/L and 87 µg/L at sites MS1 and MS3, respectively, while maximum Cu concentrations were 19 µg/L and 16 µg/L at the same sites. In Belém, contamination was dominated by Zn and Mn, with the highest levels observed in samples B2 and B3 (Fig. 2). Santarém exhibited the lowest metal contamination levels, with concentrations comparable to those found in the Tapajós River after dilution of urban effluents. This suggests limited anthropogenic pressure in Santarém, likely due to the smaller city size and reduced urban contributions to water contamination, consistent with prior studies assessing exposure levels of organic urban contaminants (Rico et al. 2021, 2022).

**Fig. 3:**
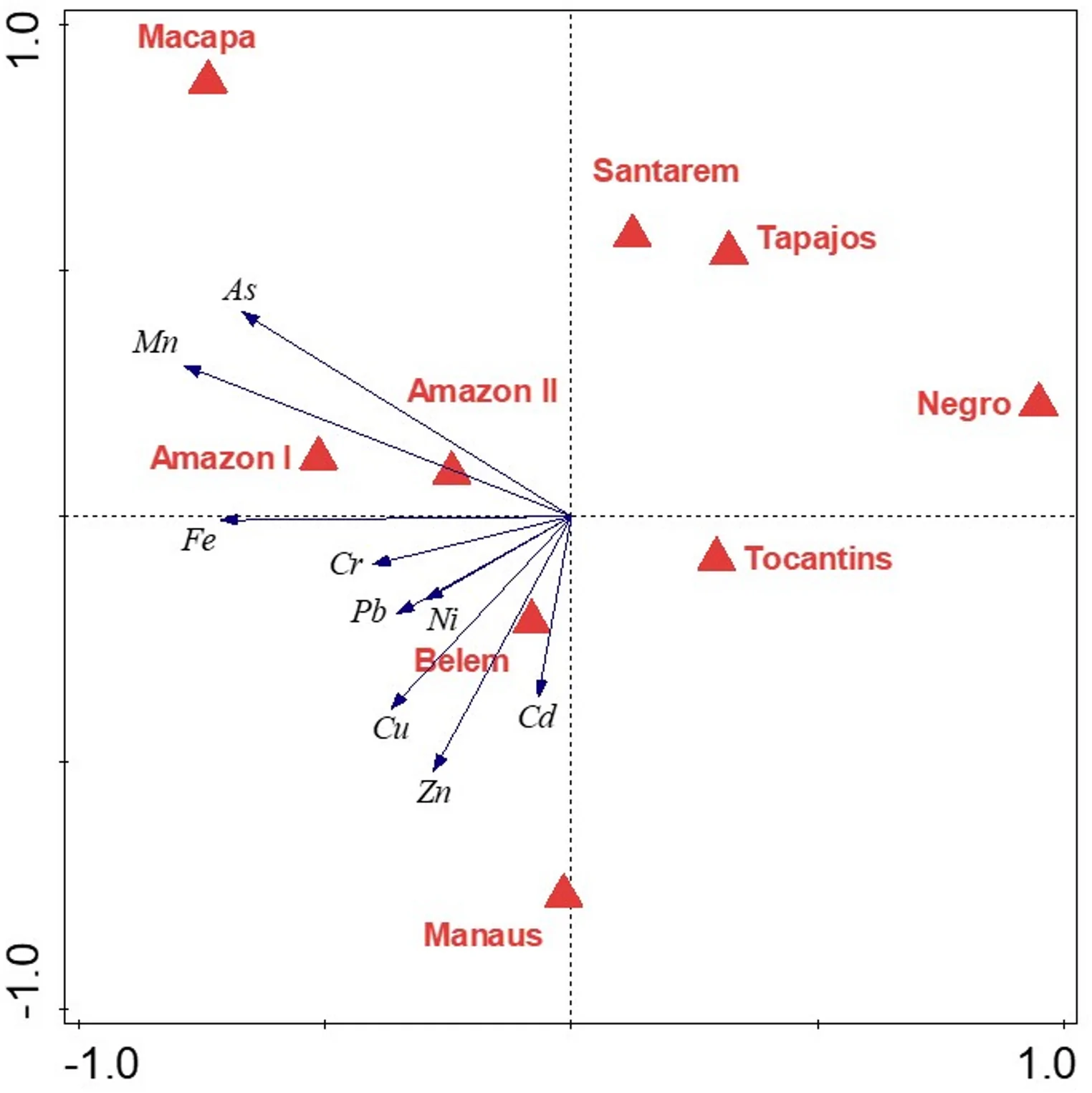
Redundancy Analysis (RDA) biplot showing the variation in total metal concentrations among sample groups. The sample groups account for 51% of the total variance, with 32% represented on the x-axis and 10% on the y-axis. The Monte Carlo p-value of 0.002 indicates significant differences in metal exposure levels between the sample groups

The RDA analysis illustrates the metal contamination profiles for different sampling groups. Along the x-axis, variability in metal concentrations among Amazon River tributaries is evident, with the Negro, Tapajós, and Tocantins rivers consistently showing lower metal levels compared to the Amazon River, which exhibited higher Mn and Fe concentrations. The y-axis highlights differences in metal concentrations between samples from streams near Manaus and Belém—characterized by elevated Zn and Cu—and those near Macapá, which showed higher Mn and As concentrations (Fig. 3).

**Fig. 4:**
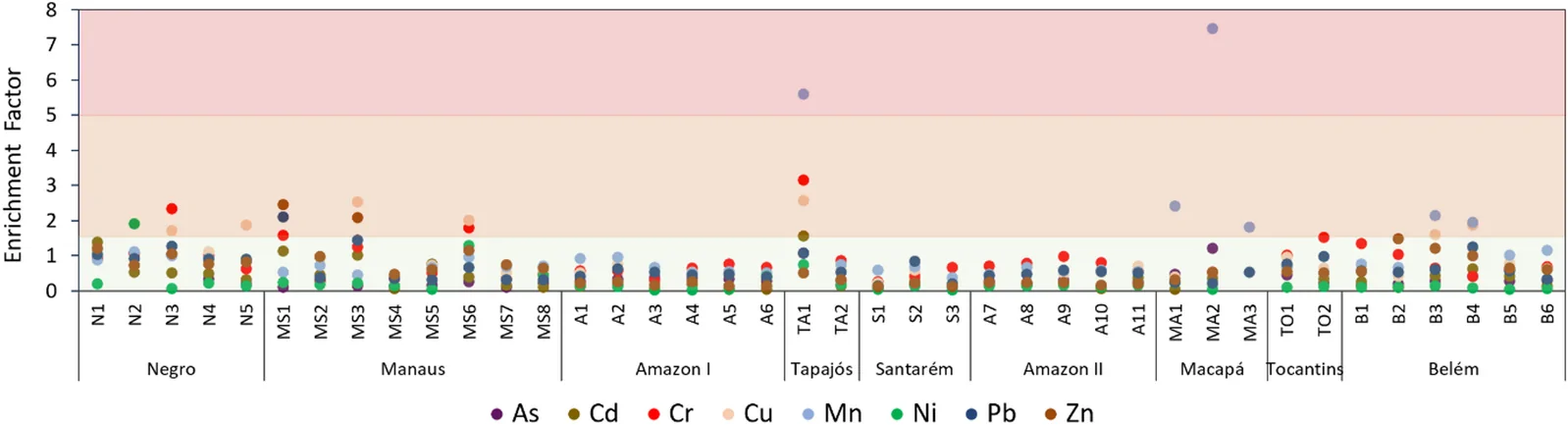
Metal Enrichment Factors (EFs) calculated based on total metal concentrations. EFs below 1.5 indicate minimal enrichment (green area), values between 1.5 and 5 indicate moderate enrichment (orange area), and values above 5 indicate very high enrichment (red area)

The analysis of EFs for metals revealed minimal enrichment in the majority of samples collected from the Amazon River and its tributaries (Fig. 4). Notable exceptions, however, were observed in the Negro River, where samples N3 and N5 exhibited moderate enrichment of Cr and Cu. These sites receive untreated urban effluents and are also subject to contamination from boat traffic, which likely explains the elevated levels. In the Tapajós River, sample TA1 showed moderate enrichment for Cu and Cr, and very high enrichment for Mn. Nevertheless, this anomalous pattern may be an analytical artefact resulting from the unusually low Fe concentration measured at that location, which could have biased the normalization calculation.

Regarding urban rivers, the highest EF value was recorded for Mn in Macapá (sample MA2, EF = 7.5), while other samples from the same city also displayed moderate Mn enrichment (Fig. 4). The precise sources of Mn in this urban setting remain unclear; however, they may be associated with iron/steel refining industries, road construction activities, or urban runoff (Rodrigues et al. 2019). In the streams of Manaus (samples MS1, MS3, and MS6), moderate enrichment of Zn, Cr, Cu, and Pb was detected, while one sample from Belém (B3) showed moderate enrichment for Cu. These patterns likely reflect a combination of industrial effluents and untreated domestic sewage originating from densely populated areas, and are consistent with previous studies that documented metal contamination in rivers across Manaus and other urban areas in the Amazon region (Gomes et al. 2023; Lages et al. 2022).

The Amazon River Basin is a major transporter of sediments and metals to the Atlantic Ocean, significantly influencing the biogeochemistry of adjacent marine environments (Constantino et al. 2019; Seyler and Boaventura 2003). Understanding these loads is crucial for assessing potential coastal and marine ecosystem impacts. Based on the Amazon River’s annual discharge of approximately 5 × 10^12^ m^2^/year (Callède et al. 2010) and the total metal concentrations measured in the Amazon II samples, we estimate an average annual metal load of 11 × 10^6^ tonnes into the Atlantic Ocean (min-max: 6–15 × 10^6^ tonnes/year), with Fe comprising 97% and Mn 1.5% of the total. Of this total load, about 44% is strongly bound to suspended particles, likely leading to deposition within the Amazon plume. It should be noted, however, that the fraction of metals bound to suspended particles may have been underestimated due to the in-situ acidification method used in our study. Conversely, the total metal load may be somewhat overrepresented, as the calculations were performed using concentrations measured during the dry season. Nevertheless, our values fell within the range of those reported in previous studies conducted across multiple seasons (Furch and Junk 1997; Benedetti et al. 2003).

**Table 3:** Metal water concentrations (median; minimum-maximum; µg/L) in the different sample groups and water quality standards set by CONAMA 357/2005 and the the European Directive 2008/105/EC.

Metal		Amazon River and major tributaries					Smaller tributaries and streams crossing urban areas				Water quality standards	
		Amazon River I (*n* = 6)	Amazon River II (*n* = 5)	Negro River (*n* = 5)	Tapajós River (*n* = 2)	Tocantins River (*n* = 2)	Manaus (*n* = 8)	Santarém (*n* = 3)	Macapá (*n* = 3)	Belem (*n* = 6)	CONAMA 357/2005	EU Directive 2008/105/EC
As	T	1.49 (1.23–1.77)	< 1.04 (< 1.04–1.16)	< 1.04 (< 0.36–<1.04)	< 1.04 (< 1.04–1.47)	< 1.04 (< 1.04–<1.04)	< 1.04 (< 0.36–<1.04)	< 1.04 (< 0.36–<1.04)	2.33 (2.07–2.59)	< 1.04 (< 1.04–<1.04)	10^a^	–
	R	0.78 (0.25–1.18)	0.66 (< 0.49–0.87)	< 0.18 (< 0.18–<0.49)	0.72 (0.59–1.10)	< 0.49 (< 0.49–0.55)	< 0.49 (< 0.18–<0.49)	< 0.49 (< 0.18–0.54)	2.09 (1.59–2.48)	< 0.49 (< 0.49–0.57)		
Cd	T	< 0.11 (< 0.03–<0.11)	< 0.11 (< 0.03–<0.11)	< 0.03 (< 0.03–<0.11)	< 0.11 (< 0.11–<0.11)	< 0.11 (< 0.11–<0.11)	0,12 (< 0.03–0.37)	< 0.11 (< 0.03–<0.11)	< 0.11 (< 0.03–<0.11)	< 0.11 (< 0.03–<0.11)	1^a^	AA: 0.08–0.25^b^ MAC: 0.5–1.5^b^
	R	< 0.05 (< 0.01–0.06)	< 0.05 (< 0.01–<0.05)	< 0.01 (< 0.01 < 0.01)	< 0.05 (< 0.05–<0.05)	< 0.05 (< 0.05–<0.05)	0.09 (< 0.01–0.36)	< 0.05 (< 0.01–<0.05)	< 0.05 (< 0.01–<0.05)	< 0.05 (< 0.01–<0.05)		
Cr	T	2.46 (< 2.24–3.39)	2.57 (1.12–3.57)	< 2.24 (< 2.24–<2.24)	2.68 (2.25–4.00)	< 2.24 (< 2.24–3.23)	2.25 (< 1.0–6.5)	< 2.24 (< 1.0–2.56)	< 2.24 (< 2.24–<2.24)	< 2.24 (< 1.0–2.45)	50^a^	–
	R	0.81 (0.5–1.18)	0.87 (0.60–1.10)	0.50 (0.34–0.84)	1.00 (0.85–1.33)	1.03 (0.97–1.08)	1.61 (0.28–4.35)	0.61 (0.37–0.83)	0.66 (0.53–0.79)	1.22 (0.46–1.98)		
Cu	T	4.59 (3.31–6.05)	3.46 (2.61–4.55)	1.47 (0.68–2.79)	3.57 (3.01–5.24)	2.3 (2.01–2.67)	6.89 (1.39–19.2)	1.80 (0.81–3.06)	1.94 (0.72–3.17)	3.62 (1.93–5.31)	9^b^	–
	R	3.43 (2.11–5.06)	2.66 (1.85–3.55)	0.75 (< 0.47–1.90)	2.76 (2.25–4.27)	1.97 (1.76**–**2.17)	6.21 **(**1.07**–18.08**)	1.30 (0.49–2.32)	1.73 (0.56–2.83)	3.02 (1.35–4.70)		
Fe	T	2778 (2324–3719)	2114 (1218–2876)	350 (293–496)	1870 (1517–2932)	1016 (691–1339)	1405 (395–2600)	1870 (1199–2433)	2008 (1303–2585)	1264 (756–1772)	300^b^	–
	R	**1363** **(836–2003)**	**1185** **(732–1636)**	280 (258**–336)**	**1107** **(902–1722)**	**765** **(599–930)**	**1164** **(320–2050)**	**1107** **(890–2078)**	**1863** **(1322–2448)**	**955** **(658–1252)**		
Mn	T	53.9 (35.2–90.4)	33.6 (17.9–52.0)	8.77 (7.69–11.3)	41.6 (36.3–57.7)	15.5 (12.4–18.6)	19.0 (10.0–36.8)	25.3 (19.0–30.8)	**179** **(108–256)**	44.6 (26.7–62.2**)**	100^a^	–
	R	46.1 (24.6–81.9)	29.2 (16.9–44.5)	8.25 (7.21–10.1)	36.8 (32.3–50.2)	14.2 (12.1–16.3)	20.8 (9.79–36.3)	22.8 (18.2–26.6)	176 (107–251)	42.9 (25.1–60.7)		
Ni	T	2.42 (< 0.83–6.11)	1.82 (1.18–2.48)	0.90 (< 0.25–3.10)	2.0 (1.70–2.98)	< 0.83 (< 0.83–0.94)	2.13 (< 0.25–3.49)	0.84 (< 0.83–1.43)	1.96 (< 0.83–3.50)	< 0.83 (< 0.83–1.10)	25^a^	AA: 4^b^ MAC: 34^b^
	R	0.85 (< 0.82–1.42)	< 0.82 (< 0.82–1.07)	< 0.24 (< 0.24–<0.24)	1.04 (0.86–1.60)	< 0.82 (< 0.82–<0.82)	1.57 (< 0.82–3.34)	0.29 (< 0.24–<0.82)	1.47 (< 0.24–2.59)	< 0.82 (< 0.82–<0.82)		
Pb	T	2.39 (1.86–3.00)	1.95 (1.17–2.52)	0.64 (0.50–0.81)	1.90 (1.56–2.92)	1.63 (0.96–2.41)	3.01 (0.37–9.93)	1.90 (0.32–2.59)	1.33 (0.53–2.20)	1.26 (0.66–1.86)	10^a^	AA: 1.2^b^ MAC: 14^b^
	R	**1.71** (1.03–**2.6**)	**1.43** (0.92–**1.94**)	0.44 (0.32–0.57)	**1.45** (1.20–**2.19**)	**1.44** (0.86–**2.03**)	**2.65** (0.33–**8.88**)	**1.45** (0.29–**2.02**)	0.96 (0.53–**1.34**)	**1.15** (0.62–**1.68**)		
Zn	T	11.5 (7.50–16.7)	9.70 (4.99–14.0)	6.41 (4.29–8.31)	19.0 (19.0–19.0)	10.8 (7.70–14.0)	43.7 (7.69–127)	5.64 (2.98–7.93)	15.6 (14.1–17.1)	28.2 (12.6–43.8)	180^a^	–
	R	7.99 (4.82–10.5)	7.74 (5.20–10.9)	4.77 (2.19–10.11)	10.9 (8.88–17.0)	10.4 (8.67–12.2)	41.4 (6.27–128)	5.33 (3.01–7.16)	13.7 (8.56–17.5)	16.1 (6.97–25.3)		

Values in bold indicate concentrations higher than at least one of the water quality standards. “Lower than” values indicate that the metal concentration was below the LOQ or LOD. T: total concentration; R: recoverable fraction; n: number of samples in each group. ^a^: water quality standard set for the total concentration in water; ^b^: water quality standard set for the dissolved fraction; AA: annual average; MAC: maximum allowable concentration. The raw data used to build this table can be found in Table S8

### Comparison with Regulatory Standards

A comparison of the measured metal concentrations against the water quality standards established by CONAMA Resolution 357/2005 (Brasil, 2005) and the EU Directive 2013/39/EU (European Commission, 2013) revealed that Fe levels exceeded the acceptable limits for environmental protection in the majority of the analyzed samples, with the exception of those collected in the Negro River (samples N1–N4; Table 3). The annual average water quality standard for Pb set by the EU Directive was exceeded in 33% of the samples, with the highest exceedance recorded in Manaus (sample MS1). Additionally, Mn concentrations surpassed the CONAMA standards in all samples from Macapá, while Cu levels exceeded the limits in two samples from Manaus (MS1 and MS3). The remaining metals analyzed in this study (As, Cd, Cr, Ni, and Zn) consistently remained below the acceptable limits (Table 3).

### Ecological Risk Assessment

The acute and chronic risk assessments identified significant ecological risks associated with Fe at nearly all sampling sites. The PAF values for Fe, estimated with the extractable metal concentration and the SSD built with acute toxicity data, ranged from 11% to 34% across sampling sites in the Amazon River and its main tributaries (except TA1, which had a value of 5%). Chronic msPAF values were even higher, ranging from 15% to 73% (Figure S1). While Fe is a naturally occurring element in Amazonian waters (Quesada et al. 2010; Seyler and Boaventura 2003), excessive enrichment—whether from natural or anthropogenic sources—can lead to oxidative stress, physiological disruption, and population-level effects (Cadmus et al. 2018; Cardwell et al. 2023). However, in the Amazon, aquatic species have likely undergone genetic adaptation to these naturally high Fe conditions (and possibly Mn) over evolutionary time. Consequently, deviations from theoretical baseline concentrations should be interpreted as indicators of environmental filtering rather than as absolute risk estimates. These deviations, therefore, serve as key drivers in shaping habitat characteristics and structuring species assemblages across the basin (Bogotá-Gregory et al. 2020).

**Fig. 5:**
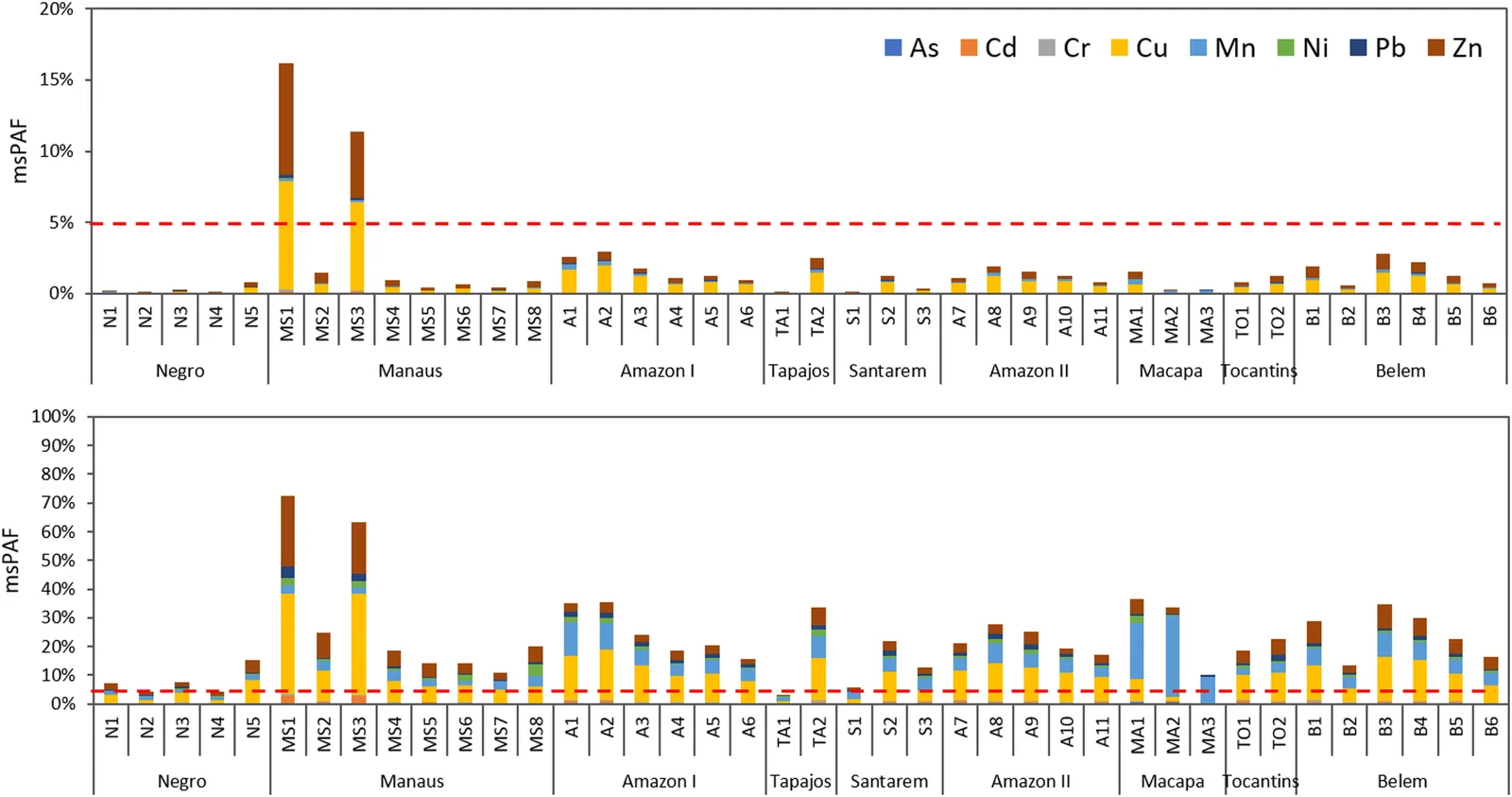
Results of the ecological risk assessment for (**a**) acute exposure and (**b**) chronic exposure (exluding Fe). The results are shown in terms of the multi-substance Potentially Affected Fraction (msPAF) of species calculated with the independent action model. The different colors represent the relative contribution of each metal to the calculated msPAF. msPAF values above 5% (dasshed line) are considered to result in high ecological risks. The PAF and msPAF values for the different metals can be found in Table S9

Excluding Fe, acute msPAF values in the Amazon River and its tributaries, calculated based on the measured metal mixtures and the independent action model were below 5% in all cases. Chronic msPAF values ranged between 3% and 36%, with Cu and Mn having the largest contribution to the calculated risk. The samples from the largest chronic msPAF corresponded to the Amazon River, and the confluence between the Tapajós river and the Amazon River (TA2; Fig. 5).

In urban areas, acute msPAF values were generally below 5%, with the exception of two samples collected from streams in Manaus MS1 and MS3, with values of 16% and 11%, primarily due to elevated levels of Cu and Zn. Chronic msPAFs urban areas were all above 5%, particularly in Manaus, with values reaching 72% and 63% in MS1 and MS3, respectively. The main contributors to chronic toxicity were Cu, Mn, and Zn followed by Pb and Ni (Fig. 5).

Exposure to these metals can induce oxidative stress, promoting the production of reactive oxygen species (ROS), a key mechanism in metal-induced cellular damage. Borković-Mitić et al. (2016) documented oxidative damage in the liver (Cu and Fe) and skin (Mn and Zn) of the freshwater amphibian *Pelophylax ridibundus*, reinforcing concerns about chronic metal exposure in aquatic organisms. Toxicity studies indicate that chronic exposure to Cu and Zn can be lethal to early life stages of fish species such as *Acipenser transmontanus* and *Oncorhynchus mykiss* at concentrations as low as 1–2 µg/L (Wang et al. 2014). Additionally, Cu has been shown to cause gill damage, while Zn disrupts enzyme activity and impairs growth (Chen et al., 2012; Ucun et al. 2021). Furthermore, excessive concentrations of these metals are linked to reproductive impairments, genotoxic and carcinogenic effects, endocrine disruption, and liver damage, potentially progressing to necrosis and mortality (Maurya et al. 2019; Singh and Sharma 2024).

Our chronic risk assessment focused on direct toxic effects on growth, reproduction, and survival, without accounting for bioaccumulation or biomagnification, which could amplify risks at higher trophic levels (Signore et al. 2016). Waichman et al. (2025) revealed significant bioaccumulation of Cd, Pb, and Hg in Amazonian carnivorous fish compared to herbivorous or omnivorous species, highlighting the role of food-chain accumulation in long-term exposure. Furthermore, their study identified Hg, Cr, and As as posing short-term health risks to the local population that depend on fish for their diet. Although their research sampled fish away from major urban areas, it suggests that fishing near urban zones with higher water exposure levels, such as those in this study, could result in greater metal uptake and bioaccumulation, rendering such practices inadvisable as feeding resources for the local population.

The application of SSDs is critical for acute and chronic toxicity assessments, providing a statistical framework to evaluate interspecies sensitivity variations and mixture toxicity effects on species assemblages (de Zwart and Posthuma 2005; Croteau et al. 2019). However, our risk calculations may have been overestimated, as they were performed using the extractable fraction obtained after acidification of the environmental samples. This concentration is expected to be higher than the dissolved concentration typically used in laboratory toxicity experiments that provide the data for constructing SSDs. Conversely, the low pH and alkalinity characteristic of Amazonian waters can increase metal bioavailability and toxicity under natural conditions. The pH of the river samples analyzed in this study was measured during the sample acidification phase. The samples were rather acidic, with pH values ranging from 4.5 to 5.5 in those collected from the Negro River, Tapajós River, and several streams near Manaus (all classified as blackwaters), and from 5.5 to 6.5 in samples from the remaining locations (mostly whitewaters). Furthermore, as reported in the literature, the alkalinity of these samples is low, falling below 0.02 meq/L in blackwaters and ranging between 0.1 and 0.8 meq/L in whitewaters, while dissolved organic carbon (DOC) concentrations are generally higher in blackwaters than in whitewaters (Gérard et al. 2003; Bogotá-Gregory et al. 2020). In blackwaters, low pH and alkalinity increase metal solubility and bioavailability, while higher DOC content can (partially) mitigate toxicity through metal complexation and, depending on the specific metal and environmental conditions, facilitate metal transport. In whitewaters, the higher pH and alkalinity promote metal precipitation and complexation with inorganic ligands, thereby reducing the concentration of free metal ions and their associated toxic potential (Duarte et al. 2024). Existing models for estimating bioavailability and toxic effects under varying environmental conditions are available for only a few metals and have been validated for a limited number of aquatic species (de Souza et al. 2023; Duarte et al. 2024). As a result, these models were not applied in this study. However, special attention should be paid to metal contamination and risks in blackwater areas where bioavailability may be potentiated (i.e., Negro River, Tapajós River and urban streams of Manaus). The background concentrations derived in this study for less impacted areas, such as the Anavilhanas National Park or sections of the Amazon River with minimal anthropogenic influence, can serve as a foundation for establishing baseline metal concentrations in both blackwaters and whitewaters, and for deriving regionally specific water quality standards.

## Conclusions

We conducted a comprehensive evaluation of metal exposure and associated ecological risks in the surface waters of the Amazon River Basin, with a particular focus on assessing the contamination burden linked to urban areas in the region. Our findings reveal widespread metal contamination in urbanized zones with limited wastewater treatment infrastructure. Fe and Pb consistently exceeded national and international water quality standards established for the protection of aquatic life. In addition, peak concentrations of Mn and Cu surpassed these thresholds in urban rivers. It is important to note, however, that Fe and Mn are naturally abundant elements in the Amazon Basin, and long-term evolutionary adaptation may have conferred tolerance to local aquatic species, reducing the direct toxicity risk associated with these metals. Our metal enrichment analysis indicates that metal contamination is spatially associated with densely populated areas. Mn was primarily enriched in Macapá, while Zn, Cr, Cu, and Pb showed enrichment in selected samples from Manaus and Belém. The probabilistic risk assessment, based on toxicity data for primary producers, invertebrates, and fish, suggests that metal mixtures are unlikely to result in acute toxicity in the Amazon River and its major tributaries. However, acute toxicity may occur in localized hotspots within large urban areas, particularly in Manaus. Chronic toxicity, on the other hand, is more likely to occur in certain areas, especially in urban settings with inadequate sanitation infrastructure, driven primarily by Cu, Mn, and Zn concentrations, and to a lesser extent by Pb and Ni. These results underscore the need for region-specific mitigation strategies, including stricter industrial discharge regulations and the implementation of advanced wastewater treatment technologies. Furthermore, our study highlights the critical importance of preserving less-impacted regions, which can serve as reference baselines for establishing regionally appropriate water quality criteria and for conserving the Amazon’s exceptional aquatic biodiversity.

## Supplementary Information

Below is the link to the electronic supplementary material.

Supplementary Material 1

## Data Availability

All data belonging to this article has been provided in the Supplementary Material file. Additional information may be requested by contacting the corresponding author of this article.
